# Interactome of *E. piscicida* and grouper liver proteins reveals strategies of bacterial infection and host immune response

**DOI:** 10.1038/srep39824

**Published:** 2017-01-03

**Authors:** Hui Li, Qing-feng Zhu, Xuan-xian Peng, Bo Peng

**Affiliations:** 1 Center for Proteomics, State Key Laboratory of Bio-Control, MOE Key Lab Aquat Food Safety, School of Life Sciences, Guangdong Province Key Laboratory for Pharmaceutical Functional Genes, Sun Yat-sen University, University City, Guangzhou 510006, People’s Republic of China

## Abstract

The occurrence of infectious diseases is related to heterogeneous protein interactions between a host and a microbe. Therefore, elucidating the host-pathogen interplay is essential. We previously revealed the protein interactome between *Edwardsiella piscicida* and fish gill cells, and the present study identified the protein interactome between *E. piscicida* and *E. drummondhayi* liver cells. *E. drummondhayi* liver cells and bacterial pull-down approaches were used to identify *E. piscicida* outer membrane proteins that bind to liver cells and fish liver cell proteins that interact with bacterial cells, respectively. Eight bacterial proteins and 11 fish proteins were characterized. Heterogeneous protein-protein interactions between these bacterial cells and fish liver cells were investigated through far-Western blotting and co-immunoprecipitation. A network was constructed based on 42 heterogeneous protein-protein interactions between seven bacterial proteins and 10 fish proteins. A comparison of the new interactome with the previously reported interactome showed that four bacterial proteins overlapped, whereas all of the identified fish proteins were new, suggesting a difference between bacterial tricks for evading host immunity and the host strategy for combating bacterial infection. Furthermore, these bacterial proteins were found to regulate the expression of host innate immune-related proteins. These findings indicate that the interactome contributes to bacterial infection and host immunity.

Edwardsiella is a Gram-negative genus of bacteria of the family Enterobacteriaceae. The genus of Edwardsiella consists of five different species, including *Edwardsiella tarda, E. hoshinae, E. piscicida, E. anguillarum* and *E. Ictaluri*^1^,^2^,^3^,^4^,^5^*. E. piscicida,* one of the most well-characterized species in the family, not only causes edwardsiallosis in fish but also causes gastrointestinal and systematic infections such as myonecrosis, septic arthritis and would infections in humans, thus presenting severe challenge to both aquaculture and environment^6^,^7^,^8^,^9^,^10^,^11^. Thus, it is especially important to understand pathogenic mechanisms to explore strategies for controlling these infections^12^,^13^,^14^,^15^,^16^,^17^. Previous reports have indicated that *E. piscicida* is most commonly found in the gastrointestinal tract, but it has also been detected in the gills, liver, spleen and kidneys of naturally and experimentally infected fish^18^,^19^,^20^, suggesting that these organs are targeted during bacterial infection. The occurrence of infectious diseases is related to the interaction between the pathogen and the host. Therefore, understanding the interaction is required for combating the pathogen. However, the interactions between the host and pathogen are largely unknown, which impedes the understanding of bacterial pathogenicity.

*E. piscicida* infects a wide variety of fish host, including grouper^21^. We recently developed a pull-down-based 1-DE proteomics approach for investigating the protein interactome between *E. drummondhayi* gill cells and *E. piscicida*, corresponding to the first step of bacterial entry. Five heterogeneous protein-protein interactions were detected, involving eight bacterial proteins and twelve fish gill proteins. Three of the eight bacterial proteins showed a protective ability in a mouse model, while all of the gill proteins were highly homologous to proteins contributing to immunity in humans and other animals^22^. These findings indicate the importance of the heterogeneous interactome. Thus, it is necessary to further explore protein interactions between this bacterial pathogen and other organs, especially susceptible internal organs, to understand bacterial pathogenicity and host immunity.

As noted above, the liver is one of susceptible internal organs infected by *E. piscicida*. Liver lesions are regarded as a typical symptom of infectious disease^18^,^19^,^20^. Therefore, investigation of the protein interactome between the liver and this pathogen is especially important for understanding of its pathogenesis. The liver contains many immunologically active cells and is responsible for immunological effects, indicating its importance in immunity. In the present study, pull-down approaches involving grouper liver proteins and bacterial cells were used to characterize the bacterial outer membrane proteins that interacted with liver proteins as well as liver proteins that bound to bacterial outer membrane proteins. Our results allowed us to generate the first heterogeneous protein interactome between *E. piscicida* envelope proteins and grouper liver cells.

## Results

### Identification of grouper liver proteins bound to *E. piscicida* using bacterial pull-down

**Figure 1:**
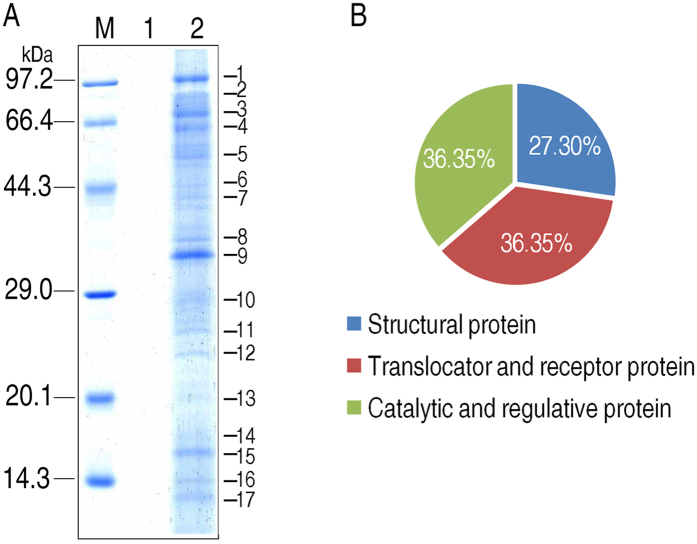
Proteomic identification of *E. drummondhayi* livers proteins pulled down by *E. piscicida* whole cell. (**A**) SDS-PAGE map for the isolation of fish liver proteins pulled-down by *E. piscicida.* M, marker; 1, Control group; 2, Test group. (**B**) The pie-chart shows the distribution of the identified liver proteins based on functional categories.

To investigate the host proteins that bind to *E. piscicida*, inactivated *E. piscicida* cells were used to pull down grouper liver proteins. Proteins that bind to bacterial surface were eluted, and resolved by electrophoresis. Seventeen protein bands were detected (Fig. 1A), where sixteen of them (except band 1) were identified through mass spectrometry. Twelve bands were identified as host proteins, which represent 11 protein entries, including immunoglobulin mu heavy chain (bands 2 and 3), flavin-containing monooxygenase (band 4), glutaryl-CoA dehydrogenase (band 6), acetyl-Coenzyme A acyltransferase (band 7), annexin max1 (band 9), cathepsin K (band 10), hyperosmotic glycine-rich protein (band 13), ribosomal protein S16 (band 14), ribosomal protein S26 (band 15), hemoglobin beta chain (band 16), serum amyloid A-5 protein precursor (band 17), and four bands were identified as bacterial proteins, incluidng dihydrolipoamide dehydrogenase (band 5), an unnamed protein product (band 8), the ahpC gene product (band 11) and iron-cofactored superoxide dismutase (band 12) (Table 1). The host proteins were categorized as structural proteins (annexin max1, ribosomal protein S16, ribosomal protein S26), translocator and receptor proteins (immunoglobulin mu heavy chain, hyperosmotic glycine-rich protein, hemoglobin beta chain, serum amyloid A-5 protein precursor), and catalytic and regulatory proteins (flavin-containing monooxygenase, glutaryl-CoA dehydrogenase, acetyl-Coenzyme A acyltransferase, cathepsin K) (Fig. 1B).

### Response of grouper liver proteins to *E. piscicida*

**Figure 2:**
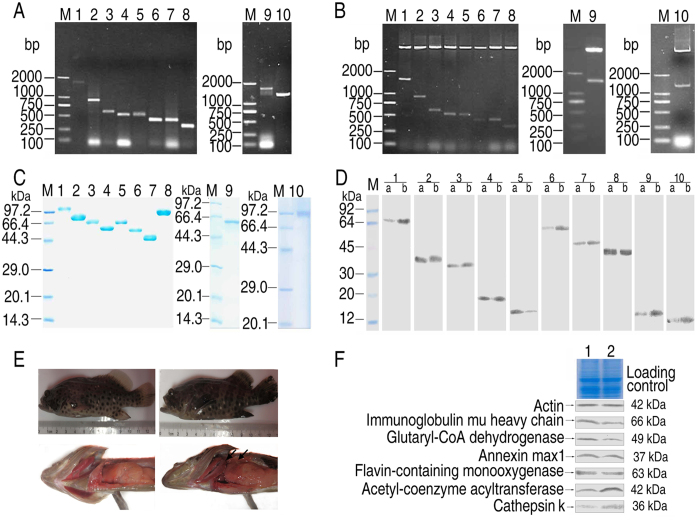
Detection of *E. piscicida*-bound liver proteins in response to *E. piscicida* infection. (**A**) PCR amplification. (**B**) Recombinant plasmids were checked via restriction endonuclease digestion. 1, *ighM* (1746 bp), 2, *acaA2* (920 bp), 3, *anxA4* (603 bp), 4, *ctsK* (534 bp), 5, *cirbP* (528 bp), 6, *rpsL6* (438 bp), 7, *hbbE*2 (444 bp), 8, *rps*26 (342 bp), 9, *fmo*5 (1683 bp), 10, *gcdH* (1326 bp), M, marker. (**C**) Purity of recombinant proteins. 1, Immunoglobulin mu heavy chain (66 + 40 kDa), 2, Acetyl-Coenzyme A acyltransferase (42 + 40 kDa), 3, Annexin max1 (37 + 40 kDa), 4, Ribosomal protein S16 (16 + 40 kDa), 5, Cathepsin K (36 + 40 kDa), 6, Hemoglobin beta chain (16 + 40 kDa), 7, Ribosomal protein S26 (13 + 40 kDa), 8, Glutaryl-CoA dehydrogenase (49 + 40 kDa), 9, Hyperosmotic glycine-rich protein (19 + 40 kDa), 10, Flavin-containing monooxygenase (63 + 40 kDa), M: marker. (**D**) Verification of the specificity of mouse antisera for liver proteins through Western blotting, using liver proteins (a) and bacterial pull-down proteins as antigens (b). 1, Immunoglobulin mu heavy chain (66 kDa), 2, Annexinmax 1 (37 kDa), 3, Cathepsin K (36 kDa), 4, Hyperosmotic glycine-rich protein (19 kDa), 5, Hemoglobin beta chain (16 kDa), 6, Flavin-containing monooxygenase (63 kDa), 7, Glutaryl-CoA dehydrogenase (49 kDa), 8, Acetyl-Coenzyme A acyltransferase (42 kDa), 9, Ribosomal protein S16 (16 kDa), 10, Ribosomal protein S26 (13 kDa), M: marker. (**E**) *E. drummondhayi* with and without bacterial infection. Left, Control; Right, Test group. Arrow indicates infectious site. (**F**) Western blotting for protein abundance. 1, Control; 2, Test group.

To investigate how these grouper liver proteins responded to *E. piscicida* infection, we first generated antibodies against these proteins. Genes encoding these proteins were cloned from species of *Epinephelus coioides* or *Danio* ORF. Expected DNA fragments were amplified from *E. coioides* cDNA or *Danio* cDNA as templates. Recombinant proteins expressed in *E. coli* were used for antibody generation in mouse. The specificity and titering of the generated antibodies were validated through Western blotting, where only single positive band at the predicted molecular masses in Western blotting were used for further studies (Fig. 2A–D).

Groupers infected with sublethal dose of *E. piscicida* showed symptoms as gill ulceration, abdominal swelling and liver congestion with a black coloration (Fig. 2E). The livers of groupers symptomized with infection were isolated for Western blotting. The abundance of the immunoglobulin mu heavy chain, glutaryl-CoA dehydrogenase, annexin max1 and flavin-containing monooxygenase were reduced, while that of acetyl-Coenzyme acyltransferase and cathepsin K was elevated (Fig. 2F).

### Identification of bacterial outer membrane proteins bound to grouper liver proteins

**Figure 3:**
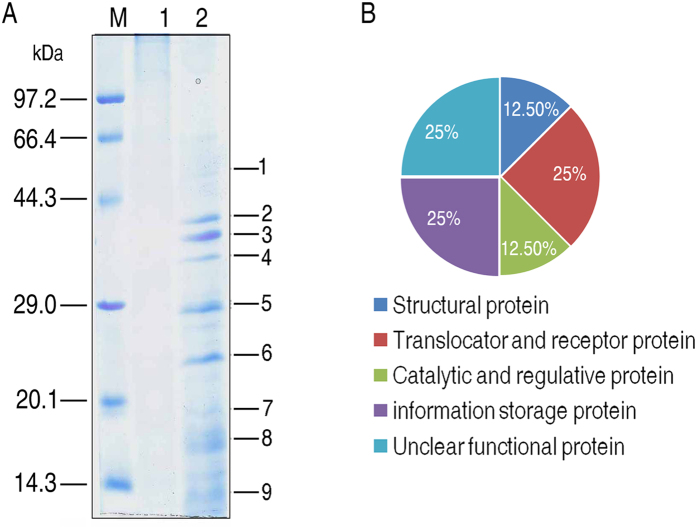
SDS/PAGE analysis of *E. piscicida* outer membrane proteins bound to fish liver proteins. (**A**) SDS/PAGE map for the isolation of outer membrane proteins pulled-down using a column containing liver protein as the affinity medium. M: marker: 1, Control; 2, Test group. (**B**) The pie-chart shows the distribution of the identified outer membrane proteins based on functional categories.

To characterize bacterial proteins involved in host and pathogen interaction, grouper liver proteins were coupled to immobilized resin. Purified *E. piscicida* outer membrane proteins were passing through the resin that retained the interacting proteins. The interacting proteins were resolved by electrophoresis followed by mass spectrometry analysis. At last, nine protein bands representing eight proteins were obtained as compared to control group (Fig. 3A). The eight proteins were ETAE_2430 (EvpB) (band 1), ETAE_1826 (band 2), ETAE_1267 (bands 3 and 4), ETAE_3048 (band 5), ETAE_0245 (band 6), ETAE_2675 (band 7), ETAE_2572 (band 8), and ETAE_0960 (band 9). Among these proteins, ETAE_1826, OmpA, ETAE_0245, ETAE_2675, ETAE_2572 and ETAE_0960 are outer membrane proteins, while EvpB and ETAE_3048 are cytoplasmic proteins (Fig. 2A; Table 2). They are classified as structural proteins (OmpA), translocator and receptor proteins (ETAE_2572, ETAE_1826), catalytic and regulatory proteins (ETAE_3048), information storage proteins (EvpB, ETAE_2675), and proteins of unclear function (ETAE_0245, ETAE_0960) (Fig. 3B).

### Response of *E. piscicida* outer membrane proteins to grouper plasma

**Figure 4:**
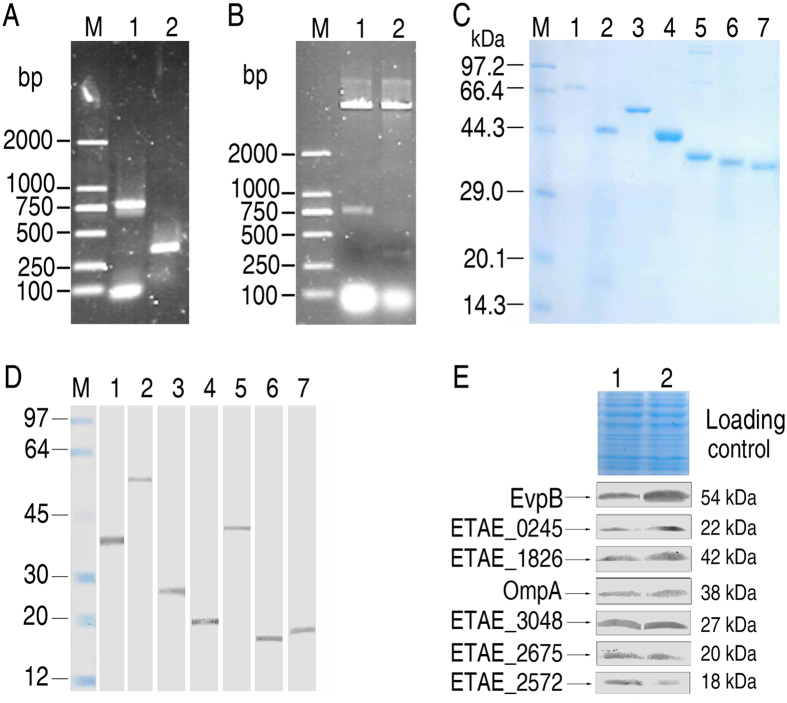
Detection of the *E. drummondhayi* liver-bound *E. piscicida* outer membrane proteins in response to exposure to fish plasma. (**A**) PCR amplification. (**B**) Recombinant plasmids were chexcked through restriction endonuclease digestion. 1, ETAE_3048 (771 bp), 2, ETAE_0960 (333 bp), M: marker. (**C**) Purity of recombinant proteins. 1, EvpB (54k + 20 kDa), 2, ETAE_1826 (42 kDa), 3, OmpA (38 + 20 kDa), 4, ETAE_3048 (27 + 20 kDa), 5, ETAE_0245 (22 + 20 kDa), 6, ETAE_2675 (20 + 20 kDa), 7, ETAE_2572 (18 + 20 kDa), M, maker. (**D**) Verification of the specificity of mouse antisera for *E. piscicida* proteins using Western blotting. 1, OmpA (38 kDa), 2, EvpB (54 kDa), 3, ETAE_3048 (27 kDa), 4, ETAE_0245 (22 kDa), 5, ETAE_1826 (42 kDa), 6, ETAE_2572 (18 kDa), 7, ETAE_2675 (20 kDa), M, marker. (**E**) Western blotting to assess outer membrane protein expression in response to exposure to *E. drummondhayi* plasma. 1, Control; 2, Test group.

We further investigated how *E. piscicida* outer membrane proteins respond to grouper plasma. Genes encoding these seven proteins were cloned from EIB202, expressed in *E. coli* and purified for antibody generation. The specificity of the prepared antibodies was validated using Western blotting. Only single stained bands were detected at the predicted molecular masses of the corresponding proteins (Fig. 4A–D).

To explore how these proteins respond to the stress imposed by host’s plasma, *E. piscicida* cells were exposed to grouper plasma. The abundance of EvpB, ETAE_0245, ETAE_1826 and OmpA was increased, whereas the expression of ETAE_3048, ETAE_2675 and ETAE_2572 was reduced (Fig. 4E). These results indicate that these outer membrane proteins are regulated by host plasma.

### Interactions between *E. piscicida* outer membrane proteins and *E. drummondhayi* liver proteins

**Figure 5:**
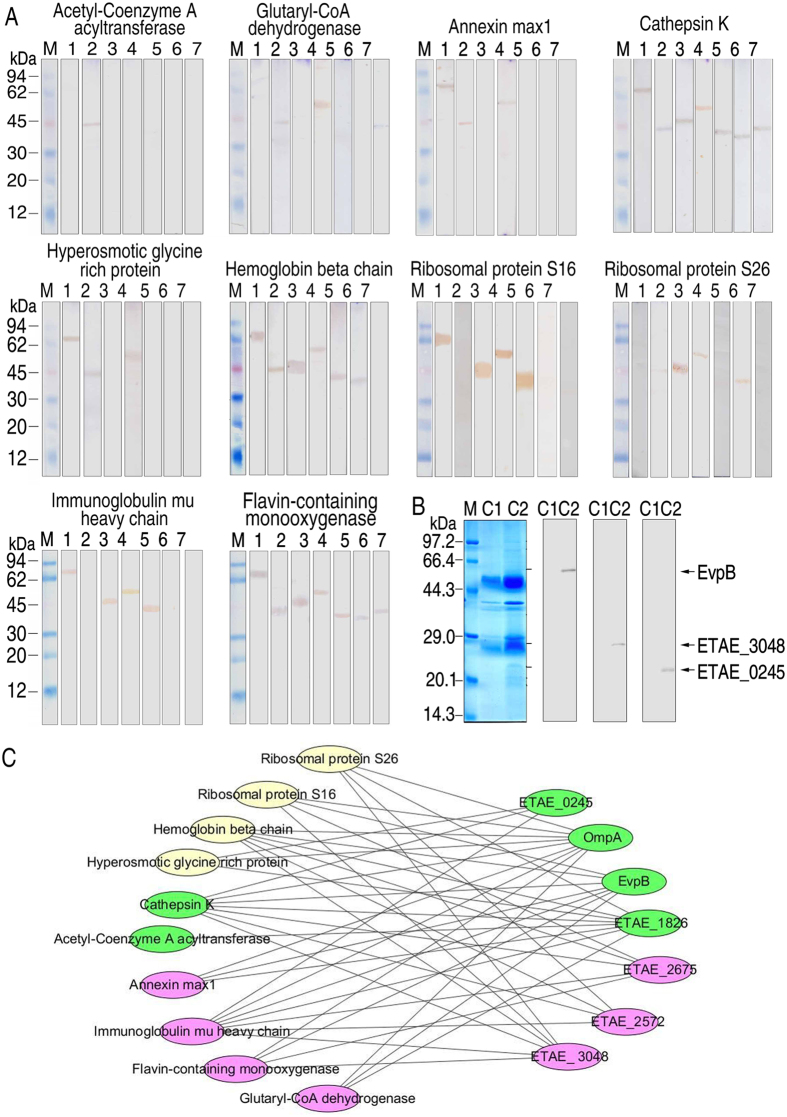
Interactions between fish liver proteins and *E. piscicida* outer membrane proteins. (**A**) The purified recombinant bacterial outer membrane proteins were transferred to NC membranes and used as bait proteins to capture prey proteins from the purified recombinant liver proteins. Then, antibodies were used to recognize the prey proteins. Bands were detected using antibodies against acetyl-Coenzyme A acyltransferase, glutaryl-CoA dehydrogenase, annexin max1, cathepsin K, hyperosmotic glycine-rich protein, hemoglobin beta chain, ribosomal protein S16, ribosomal protein S26, immunoglobulin mu heavy chain and flavin-containing monooxygenase. 1: EvpB 2: ETAE_1826 3: ETAE_3048 4: OmpA 5: ETAE_2675 6: ETAE_2572 7: ETAE_0245 (**B**) Immunoprecipitation using antiserum against cathepsin K. Bands were detected using anti-EvpB, anti-ETAE_3048 and anti-ETAE_0245. C1, Control, C2, Test group. (**C**) Network of interactional proteins. Green represents proteins upregulated under external stimuli, while red represents downregulated proteins, and yellow represents proteins for which no change was detected.

To validate the interactions between *E. piscicida* and *E. drummondhayi* liver proteins, far-Western blotting was applied. Membranes containing *E. piscicida* outer membrane proteins were incubated with the purified recombinant liver proteins individually, and then detected separately using the antibodies against acetyl-Coenzyme A acyltransferase, glutaryl-CoA dehydrogenase, annexin max1, cathepsin K, hyperosmotic glycine-rich protein, hemoglobin beta chain, ribosomal protein S16, ribosomal protein S26, immunoglobulin mu heavy chain, and flavin-containing monooxygenase. Far-Western blotting analysis showed that the liver proteins were interacting with one or more bacterial outer membrane proteins, e.g. acetyl-Coenzyme A acyltransferase interacted with ETAE_1826; glutaryl-CoA dehydrogenase interacted with ETAE_1826, OmpA and ETAE_0245; annexin max1 interacted with EvpB, ETAE_1826 and OmpA; cathepsin K interacted with all seven proteins; hyperosmotic glycine-rich protein interacted with EvpB, ETAE_1826 and OmpA; hemoglobin beta chain interacted with all of these proteins except for ETAE_0245; ribosomal protein S16 interacted with EvpB, ETAE_3048, OmpA and ETAE_2675; ribosomal protein S26 interacted with ETAE_1826, ETAE_3048, OmpA and ETAE_2572; immunoglobulin mu heavy chain interacted with EvpB, ETAE_3048, OmpA and ETAE_2675; flavin-containing monooxygenase interacted with all of the seven proteins (Fig. 5A). Furthermore, the interactions of cathepsin K to EvpB, ETAE_3048 and ETAE_0245 were confirmed by co-immunoprecipitation (Fig. 5B). Thus, the interactome between the seven bacterial proteins and ten grouper liver proteins was constructed consisting of 42 heterogeneous protein–protein interactions, as shown in Fig. 5C.

### Investigation of zebrafish, *D. rerio,* innate immune-related proteins in response to outer membrane proteins

**Figure 6:**
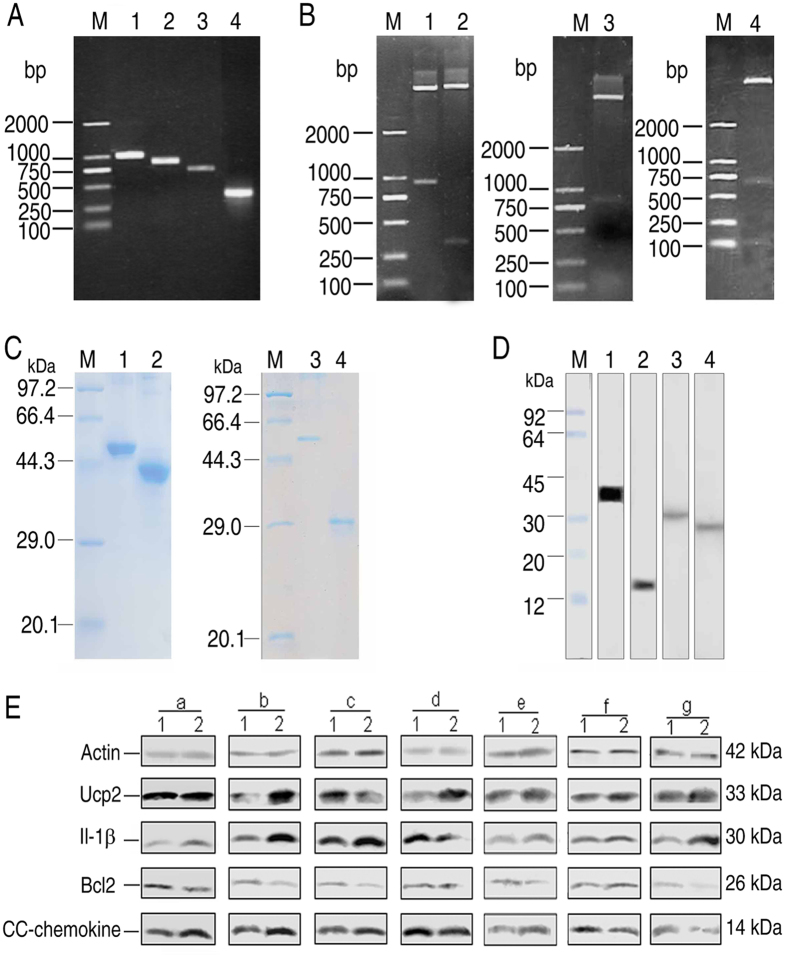
Cloning and expression of genes encoding *D. rerio* innate immune-related proteins and validation of antisera against recombinant proteins. (**A**) PCR amplification of genes encoding five *D. rerio* proteins. 1, *ucp2* (933 bp), 2, *il-1β* (822 bp), 3, *bcl*_*2*_(678 bp), 4, *cc-chemokine* (417 bp), M, marker. (**B**) Recombinant plasmids were checked via restriction endonuclease digestion. 1, *ucp2* (933 bp), 2, *cc-chemokine* (417 bp), 3, *il-1β* (822 bp), 4, *bcl*_*2*_(678 bp), M, marker. (**C**) Purity of the recombinant proteins. 1, IL-1β (30 + 20 kDa), 2, Bcl2 (26 + 20 kDa), 3, Ucp2 (33 + 20 kDa), 4, CC-chemokine (14 + 20 kDa), M, marker. (**D**) Verification of the specificity of mouse antisera against *D. rerio* proteins using Western blotting. 1, Ucp2 (33 kDa), 2, CC-chemokine (14 kDa), 3, IL-1β (30 kDa), 4, Bcl_2_ (26 kDa), M, marker. (**E**) Detection of *D. rerio* innate immune-related proteins in response to exposure to *E. piscicida* outer membrane proteins. 1, Control group, 2, Test group. Immunization with ETAE_0245 (a), ETAE_1826 (b), EvpB (c), ETAE_2572 (d), ETAE_2675 (e), ETAE_3048 (f), OmpA (g).

To investigate the role of bacterial outer membrane proteins involved in host-pathogen inteaction, *D. rerio* were randomly divided into groups and acclimatized for one week. *D. rerio* were separately injected with the recombinant outer membrane proteins ETAE_0245, ETAE_1826, EvpB, ETAE_2572, ETAE_2675, ETAE_3048 and OmpA emulsified with sterile montanide IMS 1312 VG, where the negative control group was injected with montanide IMS 1312 VG only. The body fluids of the *D. rerio* were drawn 72 hrs post-injection. the abundance of innate immune-related proteins, Ucp2, IL-1β, Bcl_2_ and CC-chemokine, were measured by Western blot. ETAE_0245 upregulated the abundance of Ucp2, IL-1β and CC-chemokine; ETAE_1826 and ETAE_2675 upregulated the abundance of Ucp2, IL-1β and CC-chemokine but down-regulated Bcl2; EvpB upregulated IL-1β and CC-chemokine but down-regulated Ucp2 and Bcl2; ETAE_2572 upregulated the level of Ucp2, and CC-chemokine but down-regulated the level of IL-1β and Bcl2; ETAE_3048 upregulated Ucp2 and Bcl2 but down-regulated CC-chemokine; OmpA upregulated the level of Ucp2 and IL-1β but down-regulated Bcl2 and CC-chemokine (Fig. 6). These results indicate that the outer membrane proteins elicit an innate immune response.

## Discussion

Bacterial pathogenesis has been extensively studied at molecular and cellular level. Many molecules have been defined during bacterial infection, e.g. toxins, effectors by type III secretions system. However, very few studies have been done at tissue level, which is rich of information regarding the interplay between host and pathogen at the onset of bacterial invasion. Thus, we considered the pathogen and tissue represents as two independent entity to invesitigate how the interplay occurred. To do that, we developed a pull-down-based 1-DE proteomics approach to characterize the heterogeneous interactome between host proteins and bacterial outer membrane proteins, representing the frontlines of interaction for both of host and pathogen. In a previous study, we investigated the interaction between *E. drummondhayi* gill cells and *E. piscicida* outer membrane proteins, and constructed an interactome with five heterogeneous protein-protein interactions^22^. However, bacterial infections involve multiple events that include entering the host, circulating in the host, and colonizing to the target organs to establish local/systematic infections. The interaction of *E. piscicida* with *E. drummondhayi* gill only represents one of them. Liver is one of the organs being targeted by *E. piscicida*. Thus, elucidating the interplay between *E. piscicida* and liver protein would gain information on the pathogensis of bacteria but anti-infection strategy by the host. To achieve this, the whole bacteria or grouper liver proteins were used as bait protein to capture grouper liver proteins or *E. piscicida* outer membrane proteins, respectively. Bound proteins were identified through mass spectrometry. To validate the interactions, far-Western blotting and Co-IP were applied. This is the first report of an interaction network between host liver proteins and bacterial outer membrane proteins.

The constructed interactome have two distinct features. First, the protein-protein interactions are not restricted to one-to-one. Some of the proteins have multiple binding targets. Second, the abudance of the interacting proteins were dynamic rather than static, implying the active involvement of these proteins in bacterial infection or host anti-infection, e.g. 1) interactions of increased host proteins with increased outer membrane proteins, such as acetyl-Coenzyme A acyltransferase with EvpB; 2) interactions of increased host proteins with decreased outer membrane proteins, such as cathepsin K with ETAE_2675; 3) interactions of decreased host proteins with increased outer membrane proteins, such as immunoglobulin mu heavy chain with EvpB; 4) interactions of decreased host proteins with decreased outer membrane proteins, such as immunoglobulin mu heavy chain with ETAE_2675. These results indicate that the heterogeneous interaction proteome may contribute to bacterial invasion and host immunity as a result of immunity or infection through these interactions.

Compared to the interactome of *E. drummondhayi* gill and *E. piscicida* outer membrane proteins^22^, some of proteins involved in grouper liver proteins and *E. piscicida* outer membrane proteins were identical. This feature implied that host might mount strategies involving different sets of proteins to eliminate bacterial pathogens in a tissue-specific manner. In the contrast, pathogens like *E. piscicida* use several key proteins to invade the host. In this study, ETAE_0245, ETAE_1826 (OmpS2), ETAE_2675 and OmpA are the key proteins by *E. piscicida* to interact with both of the grouper gill and liver. However, Pnp, EvpB, FliC and OmpF2 were only present in *E. piscicida*-grouper gill interactome, and EvpB, ETAE_2572 and ETAE_3048 were only detected in grouper liver-*E. piscicida* interactome. Of notice, ETAE_0245, OmpS2, and OmpA were identified as efficient protective immunogens^16^,^17^,^23^, suggesting that these shared outer membrane proteins may be good vaccine candidates.

Interestingly, several of the outer membrane proteins, OmpA, ETAE_1826 and EvpB, identified in the present study were not directly linking to *E. piscicida* infection. *E. piscicida* OmpA was detected in the outer membrane protein fraction using a two-dimensional electrophoresis-based proteomics approach and was shown to present immunogenic potential^24^. ETAE_1826 exhibits high homology to OmpS2, which is essential for a disseminated infection caused by pathogenic *E. piscicida*^25^. EvpB is a component of the type VI secretion system in *E. piscicida* and is vital for *E. piscicida* pathogenesis^26^. On the other hand, several of liver proteins are active in grouper immune response. The immunoglobulin mu chain is an indicator for the physiological maturity of the immune system, and more importantly, is the main immunoglobulin responsible for humoral adaptive immunity in most teleost fish^27^,^28^. Flavin-containing monooxygenase converts trimethylamine into trimethylamine oxide through an oxidation reaction, which is associated with LPS-induced inflammation^29^. Deficiency of glutaryl-CoA dehydrogenase causes type I glutaric acidemia, usually triggered by childhood infection^30^. The annexins are Ca^2+^-dependent phospholipid-binding proteins involved in many cellular processes, whose overexpression were observed in patients infected with *Helicobacter pylori* and channel catfish infected with *Edwardsiella ictaluri*^31^,^32^. Hyperosmotic glycine-rich protein contributes to the regulation of ion transfer in rainbow trout^33^. Hemoglobin β chain is involved in the stress response to changes in the environment^34^. Serum amyloid A-5 SAA is a common acute-phase protein and is activated in response to various sources of stress in fish^35^. Although the roles of these proteins in fish immunity have been well documented, our study neverthelessly expand those studies by providing the evidence that bacteria could directly interact with these proteins, which may trigger immune response.

The expression of the *D. rerio* innate immune-related proteins Ucp2, IL-1β, Bcl_2_ and CC-chemokine was adjusted in response to the presence of bacterial outer membrane proteins, indicating that these bacterial proteins are important in inducing grouper immune response. Ucp2 is mitochondrial carrier protein, which controls immune cell activation and the production of mitochondrial reactive oxygen species, cytokines and nitric oxide^36^,^37^. Interleukin-1β is a critical cytokine associated with inflammation^38^,^39^. Bcl2 regulates outer mitochondrial membrane channel (VDAC) opening, which modulates the mitochondrial membrane potential and, thus, controls the production of reactive oxygen species (ROS) and the release of cytochrome C by mitochondria. Of notice, both of Bcl2 and cytochrome C are the potent inducers of cell apoptosis^40^. CC-chemokine prompts blood cell supplementation and activation when inflammation occurs in the body, which is an important component of fish innate immunity^41^. The alteration of these immune-related molecules strongly suggested that the bacterial outer membrane proteins are the potential antigens triggering immune response, thus being vaccine candidates. Immune response of fish to outer membrane proteins isolated from species of *Edwardsiella* have been reported^42^,^43^, where pooled outer membrane proteins were used instead of recombinant proteins. Although 20 μg and 12.5–100 μg outer membrane proteins were used in 40.2 g of *Labeo robita* and 16 g of *Ictalurus punctatus*, respectively, to achieve optimal immune efficiency, we and others found that 1.5 μg of recombinant proteins are the optimal dose for 0.3–0.4 g of *D. rerio* to generate effective immune response^44^. Thus, these results suggest that dose of immunogens is relatd to fish species.

In conclusion, our study of establishing grouper liver protein-*E. piscicida* interactome has at least two important implications. First, as compared to our previous grouper gill-*E. piscicida* interactome, different host organs may adopte different strategies against the same pathogen, while bacteria may have several key proteins during infection. Second but not last, several of the outer membranes proteins were recognized by the host would trigger immune response, signifying the importance of these proteins in the initation of immune response by the host. Therefore, these proteins would be tested for their vaccine ability in future studies.

## Methods

### Sources of fish and fish housing

Approximatly two-month old *Epinephelus coioides* (body weight = 40.16 ± 3.41 g and body length = 10.22 ± 0.46 cm) were obtained from the Guangdong Daya-Bay Fishery Development Center, Huizhou, Guangdong, China. *Epinephelus coioides* were reared indoors in aquaria for disease research, where no disease outbreak had occurred during the course of breeding. Before experiment, *Epinephelus coioides* were stocked indoor at 25 °C and a density of 20 *Epinephelus coioides* per 120 L tank with a constant flow of filtered natural seawater and fed twice daily (7:00 and 18:00) to satiation with a commercially available dry diet (Guangdong Yuequn Ocean Biological Research Development Co., Ltd., Jieyang, China) for two weeks and were demonstrated to be free of *E. piscicida* through microbiological and PCR detections. Ten fish were grouped.

Three-month zebrafish (*Danio rerio*, average body length about 3 cm and average body weight about 0.3 g) were commercially obtained from Guangzhou Fangcun Huadiwan Flower Bird Fish & Insect market, Guangzhou, China. These fish were fed in 25 L opening-circuit filtered aquatic tank at room temperature with aeration and had to acclimatize to the condition of laboratory for 7 days before experiments. The keeping and treatment of the experimental fish were approved by Sun Yat-sen University.

### Ethics statement

All work was conducted in strict accordance with the recommendations of the Guide for the Care and Use of Laboratory Animals of the National Institutes of Health. The protocol was approved by the Institutional Animal Care and Use Committee of Sun Yat-sen University (Animal Welfare Assurance Number: I6).

### Infection of groupers with *E. piscicida*

The bacterial strain used in current study was *E. piscicida* EIB202, whose complete genome sequence is available^45^. To propagate the bacterium, a single colony was inoculated to 5 mL of TSB medium, followed by shaking at 30 °C for 24 h at a speed of 200 rpm. Bacterial culture was collected through centrifugation at 6,000 g for 5 min at 4 °C, and was washed twice with 25 mL saline solution. The resulting bacterial cells were resuspended with saline solution and adjusted to OD_600_ 1.0. For bacterial infection, twenty groupers were anesthetized by immersion in 100 ng/mL of tricaine methanesulphonate (MS-222, Sigma, USA), and were infected with EIB202 via intraperitoneal injection (50 μL 8 × 10^7^ cells each), where the same volume of saline solution was used as a control. Fourty-eight hours post infection, *E. drummondhayi* were anesthetized similarily as described aboved for liver isolation, which follows the previously described protocol^46^. Both of the livers from dying *E. drummondhayi* and healthy *E. drummondhayi* were isolated immediately after anesthetia for protein preparation.

### Isolation of grouper liver proteins

Liver proteins were prepared as described previously with a few modifications^46^. Briefly, five liver tissues were freshly collected from groupers, *E. drummondhayi,* and then washed in 0.85% sterile saline solution three times. Liquid nitrogen was added, followed by grinding in a Dounce tissue grinder. Next, 1 mL of protein extraction buffer (66 mmol/L Tris/HC1, pH7.2, 3% (v/v) NP40, 0.1 mmol/L PMSF) was added, and the samples were placed on in ice for 30 min. Finally, the supernatants were harvested via centrifugation of 8,000 g at 4 °C for 20 min, and the protein concentration in the supernatants was determined using the Bradford method^47^.

### Isolation of bacterial outer membrane proteins through lauryl sarcosinate extraction

Bacterial outer membrane proteins were prepared as described previously^39^. Briefly, a single colony was propagated in TSB medium at 30 °C for 24 h. The cultures were then diluted 1:100 using fresh TSB medium and grown to an OD600 of 1.0 at 30 °C. The bacterial cells were harvested via centrifugation at 6,000 g for 5 min at 4 °C. The bacterial pellet was washed in sterile saline buffer (0.85% NaCl) three times, then re-suspended in sonication buffer (50 mM Tris/HCl, pH 7.4) and disrupted through intermittent sonic oscillation of the power output 60% for 7 s with intervals of 7 s on ice for a total of 40 min. Unbroken cells and cellular debris were removed via centrifugation at 6,000 g for 15 min at 4 °C. The turbid supernatant was subjected to ultracentrifugation at 100,000 g for 1 h at 4 °C. The pellet was solubilized with 2% (w/v) sodium lauryl sarcosinate (Sigma) at 4 °C and incubated at room temperature for 40 min. Following ultracentrifugation at 100,000 g for 1 h at 4 °C, the resulting pellet was dissolved in sterile pure water (approximately 5 mg/mL) and stored at −80 °C until use.

### *E. piscicida* pull-down assay for isolation of *E. drummondhayi* liver proteins

The bacterial pull-down assay was performed as described previously^48^. *E. piscicida* EIB202 was grown overnight at 30 °C in a shaker bath. A fresh overnight culture seed was then inoculated into TSB medium (1% (w/v) peptone, 0.5% (w/v) yeast extract, 1% (w/v) NaCl, pH 7.4), cultured at 30 °C and grown to an OD600 of 1.0. The cultures were harvested through centrifugation at 6,000 g for 5 min at 4 °C and washed three times via resuspension in 0.85% (w/v) NaCl. These bacterial cells were then suspended in 1% oxymethylene (w/v), inactivated at 80 °C for 90 min and centrifuged at 6,000 g for 5 min at 4 °C. The pellet was resuspended in 0.05 M Tris–HCl (pH 8.0) including 4.5 M urea under gentle rotation for 4.5 h, followed by harvesting through centrifugation at 6,000 g for 5 min at 4 °C. After washing three times via resuspension in 0.85% NaCl, the bacterial pellet was mixed with either 2 mg *E. drummondhayi* liver proteins, as the treatment group, or 0.85% NaCl, as a negative control. The mixture was incubated for 1 h, followed by harvesting at 6,000 g for 5 min at 4 °C after being washed three times through resuspension in 0.85% NaCl. The pellet was resuspended in 0.05 M Tris–HCl (pH 8.0) containing 4 M urea, under gentle rotation for 1 h, and the proteins were harvested via centrifugation at 8,000 g for 5 min at 4 °C. The pellet was subsequently concentrated in a three-fold volume of acetone for 12 h at −40 °C, collected via centrifugation at 8,000 g for 10 min at 4 °C and then resuspended in 20 μL of 0.05 M Tris–HCl. The isolation was repeated twice.

### Sepharose 4B-bound *E. drummondhayi* liver proteins for isolation of *E. piscicida* outer membrane proteins

Cyanogen bromide Sepharose 4B 0.5 g was sufficiently expanded in 1 mM HCl for 15 min, then added to the column and washed with 50 mL of 1 mM HCl. After washing with 20 mL of binding buffer, liver proteins were added, followed by shaking for 2.5 h. The column was then washed to remove free liver proteins and blocked with 0.1 M Tris-HCl for 3 h. Following balancing using a 10-fold volume of PBS buffer, 1 mL (1 μg/μL) outer membrane proteins were added, and the column was incubated for 2 h. A 10-fold volume of PBS buffer and an equal column volume of eluted buffer were used to wash and elute the unbound and bound outer membrane proteins, respectively. The elutes were centrifuged at 8,000 g for 5 min at 4 °C. The resulting bound proteins were concentrated in acetone at −40 °C for 12 h, then collected through centrifugation at 8,000 g for 10 min at 4 °C and resuspended in 0.05 M Tris–HCl. The isolation was repeated twice.

### SDS-PAGE and mass spectrometric (MS) analysis

A discontinuous electrophoresis buffer system involving Laemmli buffer, 4% stacking gels and 12% resolving gels was used to separate the proteins. All samples were boiled for 5 min after the addition of sample loading buffer and subsequently electrophoresed at a constant voltage of 120 V for the resolving gels, until the tracking dye (bromophenol blue) reached the bottom of the gels. Protein bands were visualized by staining with Coomassie Brilliant Blue R-250. The resultant bands were excised from gels and digested with trypsin through a routine procedure^49^. The sample solution (30–100 ppm), together with equivalent matrix solution, was applied to the MALDI TOF-Target system using HCCA as a MALDI matrix for peptide mapping and was prepared for MALDI-TOF/MS analysis. MALDI-TOF spectra were calibrated using trypsin autolysis peptide signals and matrix ion signals. Proteins with low confidence were further identified using MALDI TOF/TOF. For MS/MS spectra, the five most abundant precursor ions per sample were selected for subsequent fragmentation, and 1000–1200 Da laser shots were accumulated per precursor ion. The criterion for precursor selection was a minimum S/N of 50. All MALDI analyses were performed with a fuzzy logic feedback control system (Reflex III MALDI-TOF system, Bruker) equipped with delayed ion extraction. Both the MS and MS/MS data were interpreted and processed using Flexanalysis 3.0 (Bruker Daltonics), after which the obtained MS and MS/MS spectra per spot were combined and submitted to the MASCOT search engine (V2.3, Matrix Science, London, U.K.) by Biotools 3.1 (Bruker Daltonics) and subjected to searches using the following parameters: NCBI in SwissProt (http://www.matrixscience.com), one missed cleavage site, carbamidomethyl as a fixed modification of cysteine and oxidation of methionine as a variable modification, MS tolerance of 100 ppm, MS/MS tolerance of 0.6 Da. Known contaminant ions (keratin) were excluded. The subcellular locations of the proteins were determined using the program PSORTb version 2.0 (http://www.psort.org/psortb/). Protein bands from the repeated experiments were used for MS analysis and the same results were obtained.

### Cloning of genes, purification of recombinant proteins and preparation of antisera

Standard PCR and molecular biology protocols were used to amplify the *ighM, acaA2, anxA4, ctsK, cirbp, rps16, rps26, hbbE2, fmo5, gcdH* genes of *E. drummondhayi,* and *ucp*2, IL-1*, bcl*2 and CC-chemokine genes of *D. rerio* and ETAE_3048, ETAE_0960 genes of *E. piscicida*. Primers for these genes were designed according to the *Epinephelus coioides,* or *Danio* or *E. tarda* EIB202 ORF sequences released by GenBank (Supplementary Table 1). PCR fragments were detected through agarose electrophoresis and were directionally cloned into the pMAL-c2X plasmid for fish genes and pET-32a for *E. piscicida* genes. Recombinant plasmids were checked via digestion with restriction endonucleases and transformed into *E. coli* BL21. Overnight cultures of *E. coli* BL21 harboring recombinant plasmids were diluted 1:100 (v/v) in fresh LB containing ampicillin (100 μg/mL), then incubated at 37 °C until the absorbent optical density reached 0.6 at 600 nm (OD600). Protein expression was induced with 1 mM isopropyl-β-D-thiogalactoside (IPTG, from BBI) for 5 h at 37 °C after the optimization of expression conditions, including the culture temperature, IPTG concentrations and IPTG-induced period. Bacterial cells were harvested through centrifugation at 10,000 g for 20 min at 4 °C and washed with 0.85% NaCl, then resuspended in 50 mM sodium phosphate buffer (pH 8.0) containing 8 M urea and incubated for 30 min in ice bath. The cell suspension was disrupted via sonication in an ice bath (350 W, 3 × 10 min), followed by centrifugation at 12,000 g for 20 min at 4 °C. The clarified supernatant was loaded into a column packed with Ni^2+^ nitriloaceate, which was charged with 50 mM NiSO_4_, and purified through affinity chromatography on Ni-NTA super-flow resin according to the manufacturer’s instructions (Qiagen, Germany). The concentrations of the proteins were determined via the Bradford method. Solutions were stored at −80 °C until use. Antisera against the purified recombinant proteins were raised separately by immunizing mice with 100 μg of purified protein emulsified with Freund’s complete adjuvant. The first injection was followed by two other injections with Freund’s incomplete adjuvant at intervals of two weeks. Sera were collected and stored at −80 °C until use.

### Exposure of *E. piscicida* to fish plasma

The exposure of bacterial samples to stress caused by fish plasma was carried out as described previously^50^. Briefly, 4 mL of OD600 1.0 *E. piscicida* EIB202 cells were collected and washed with saline solution. Then, 200 μL of *E. drummondhayi* plasma or saline solution was added in the test and control groups, respectively. The mixtures were incubated at 37 °C for 2 h following resuspension, after which the cells were prepared for Western blotting.

### Western blotting and far-Western blotting analyses

The two assays were performed as described previously^51^,^52^. Mouse antisera against the fish immunoglobulin mu heavy chain, acetyl-Coenzyme A acyltransferase, annexin max1, ribosomal protein S16, cathepsin K, hemoglobin beta chain, ribosomal protein S26, glutaryl-CoA dehydrogenase, hyperosmotic glycine-rich protein and flavin-containing monooxygenase and against *E. piscicida* EvpB, ETAE_1826, OmpA, ETAE_3048 ETAE_0245, ETAE_2675 and ETAE_2572 were used as the primary antibodies, and a horseradish peroxidase (HRP)-conjugated rabbit anti-mouse antibody was used as the secondary antibody. For Western blotting, proteins separated from gels were transferred to 0.22 μm nitrocellulose (NC) membranes at a constant voltage of 80 V for 1 h at 4 °C, and membranes were stained with Ponceau S to evaluate the transfer efficiency. The membranes were then blocked overnight in 5% non-fat milk in Tris–NaCl-Tween (TNT) buffer at 4 °C. After rinsing three times for 15 min with TNT buffer, the membranes were separately incubated with mouse antibodies for 2 h on a gentle shaker at room temperature. The membranes were subsequently rinsed again and then incubated with rabbit anti-mouse horseradish peroxidase (HRP)-conjugated secondary antibodies for 2 h under the same conditions. The membranes were washed and developed with a dimethylaminoazobenzene (DAB) substrate system until the appearance of maximum color. All of the primary antibodies were diluted 1:100-200 in blocking buffer, and the secondary antibodies were diluted to 1:2000.

For far-Western blotting, the purified recombinant bacterial outer membrane proteins were transferred to NC membranes and used as bait proteins to capture prey proteins (purified recombinant liver proteins). The membranes were then washed with TNT and incubated with the same primary and secondary antibodies used in the Western blotting assay, after which they were developed with the DAB system as described above.

### Coimmunoprecipitation (Co-IP)

Co-IP was carried out as described previously^53^. Recombinant cathepsin K and *E. piscicida* outer membrane proteins were incubated at room temperature for 2 h on a gentle shaker. Then, 10 μL of mouse antiserum against cathepsin K was added to the test group, and pre-immune antiserum was added to the control. After incubation under the same conditions, 20 μL of nProtein A Sepharose 4 Fast Flow (Amersham Biosciences Corp.) was added, followed by incubation for 12 h at 4 °C on a gentle shaker. The nProtein A Sepharose 4 Fast Flow was collected via centrifugation at 3000 g for 5 min and cleaned six times for 10 min each with pH 7.0 Tris-HCl buffer, followed by incubation in 50 μL of 1 mM pH 2.4 glycine-HCl buffer for 2 h at room temperature. After centrifugation at 3,000 g for 5 min, the supernatant was employed for Western blotting using anti-EvpB, -ETAE_3048 and –ETAE_0245 as the primary antibodies.

### Innate immune response to recombinant outer membrane proteins

Vaccination was carried out as described previously^44^. *D. rerio* were acclimatized for one week and randomly divided into groups, ten each. These animals were intramuscularly injected injected with the recombinant outer membrane proteins (1.5 μg per fish) ETAE_0245, ETAE_1826, EvpB, ETAE_2572, ETAE_2675, ETAE_3048 and OmpA emulsified with sterile montanide IMS 1312 VG (Seppic, France), where the negative control group was injected with montanide IMS 1312 VG only. No any toxicity effect on fish was observed. Humoral fluid was collected after 3 days and used for analysis of innate immunity through Western blotting.

## Additional Information

**How to cite this article**: Li, H. *et al*. Interactome of *E. piscicida* and grouper liver proteins reveals strategies of bacterial infection and host immune response. *Sci. Rep.* **7**, 39824; doi: 10.1038/srep39824 (2017).

**Publisher's note:** Springer Nature remains neutral with regard to jurisdictional claims in published maps and institutional affiliations.

## Supplementary Material

Supplementary Tables

## Figures and Tables

**Table 1:** Identification of protein bands isolated from liver cells that bound to E. piscicida*.

Spot No.	NCBI No.	Protein description	Gene name (*Danio*)	Pred.MW (kDa)	No. of peptides matched (MS/MS)	Sequence coverage (%)	NCBI score
2	Host gi|62255676	immunoglobulin mu heavy chain	*ighM*	66	8	15	179
3	gi|62255676	immunoglobulin mu heavy chain	*ighM*	66	8	15	179
4	gi|186926668	flavin-containing monooxygenase	*fmo5*	63	4	9	260
6	gi|209155496	Glutaryl-CoA dehydrogenase	*gcdH*	49	14	41	224
7	gi|334362378	acetyl-Coenzyme A acyltransferase	*acaA2*	42	6	25	270
9	gi|328677115	annexin max1	*anxA4*	37	7	38	343
10	gi|334362475	cathepsin K	*ctsK*	36	11	66	214
13	gi|221048043	hyperosmotic glycine rich protein	*cirbp*	19	7	34	92
14	gi|335955192	ribosomal protein S16	*rps16*	16	10	49	238
15	gi|197725736	ribosomal protein S26	*rps26*	13	7	53	108
16	gi|334362285	hemoglobin beta chain	*hbbE2*	16	13	86	596
17	gi|226443107	Serum amyloid A-5 protein precursor	*saa*	13	13	32	92
	Bacterium						
5	gi|304558066	Dihydrolipoamide dehydrogenase	*dld*	51	14	40	309
8	gi|269138490	unnamed protein product		34	29	82	880
11	gi|269138312	*ahpC* gene product	*ahpC*	22	10	52	557
12	gi|112419723	iron-cofactored superoxide dismutase	*sodB*	18	4	46	286

*Reliability of these proteins was further validated by Western blot using these bound proteins as antigens in Fig. 2D(b).

**Table 2:** Identification of protein bands isolated from *E. piscicida* outer membrane proteins that bound to liver proteins*.

Spot no.	NCBI No.	Protein name	Gene name	Pred.MW (kDa)	Sequence coverage (%)	Peptides matched	NCBI score
1	gi|269139775	EvpB	ETAE_2430 (*evpB*)	54	17	8	183
2	gi|269139173	Outer membrane protein (porin)	ETAE_1826	42	56	16	617
3	gi|239758178	*ompA* gene product	ETAE_1267 (*ompA*)	38	43	16	508
4	gi|269138621	*ompA* gene product	ETAE_1267 (*ompA*)	38	44	13	464
5	gi|269140391	N-acetylmuramoyl-L-alanine amidase	ETAE_3048	27	57	15	416
6	gi|269137603	Hypothetical protein	ETAE_0245	22	83	13	450
7	gi|269140020	Virulence-related outer membrane protein	ETAE_2675	20	62	11	465
8	gi|269139917	Peptidoglycan-associated outer membrane lipoprotein	ETAE_2572	18	56	10	355
9	gi|269138316	Preprotein translocase subunit YajC	ETAE_0960	12	37	3	190

*Reliability of these proteins was further validated by Western blot using these bound proteins as antigens in Fig. 4D.
