# Supplementary material for: Interactome of E. piscicida and grouper liver proteins reveals strategies of bacterial infection and host immune response
Source: Sci Rep. 2017 Jan 3;7:39824. doi: 10.1038/srep39824 (PMC5206647; doi:10.1038/srep39824)
Supplement: Supplementary Tables [file srep39824-s1.pdf]

Interactome of *E. piscicida* and grouper liver proteins reveals strategies of bacterial infection and host immune response

Hui Li, Qing-feng Zhu, Xuanxian Peng, Bo Peng*

Supplementary Table 1 Primer information

Gene		Primer sequence（5’ - 3’）	Vector	bp	Res.site	MW
Epinephelus coioides						
ighM	Sense	5' TTGGGATCCATGTTCTCTGTAGCTCTGC 3'	c2X	1746	*BamH*I	66
	Antisense	5' TCCAAGCTTCTACTGGGCCTTGCACGTT 3'			*Hind*III	
acaA2	Sense	5' CCCGAATTCATGGCGCTTATCAGAGG 3'	c2X	920	*EcoR*I	42
	Antisense	5' CCCTCTAGACGGTTTTTCAAGAGCT 3'			*Xba*I	
anxA4	Sense	5'GGGGAATTCATGGCAGCGATCGGAAACC3'	c2X	603	*EcoR*I	37
	Antisense	5'GGGGTCGACCACACAAAGCACCGTGAGG3'			*Sal*I	
ctsK	Sense	5' GGAGAATTCGAGAAGACAGGTCAGCT 3'	c2X	534	*EcoR*I	36
	Antisense	5' CTGGTCGACTCACATGACGGGGAAG 3'			*Sal*I	
cirbp	Sense	5' GGGGAATTCATGTCGGACGAAGGTA 3'	c2X	528	*EcoR*I	19
	Antisense	5' CGGGTCGACTTACTCTGTAGCATAG 3'			*Sal*I	
rps16	Sense	5' GGGGAATTCATGCCGGCTAAAGGTC 3'	c2X	438	*EcoR*I	16
hbbE2	Sense	5' GCGGAATTCATGGTCGAATGGACAG 3'	c2X	444	*EcoR*I	16
	Antisense	5' TTCGTCGACCTAGTGGTACTGCCTT 3'			*Sal*I	
	Antisense	5' GGGGTCGACACGGTAAGACTTCTGGTA 3'			*Sal*I	
rps26	Sense	5' CCCGAATTCAGAAGAGAAGGAATAACG 3'	c2X	342	*EcoR*I	13
	Antisense	5' CCCGTCGACCTTACATGGGCTTTGG 3'			*Sal*I	
fmo5	Sense	5' GGGGAATTCATGGCTAAACGTGTTGCTAT3'	c2X	1683	*EcoR*I	63
	Antisense	5'GGGAAGCTTTCACTGTGTGGGCAATGGTA3'			*Hind*III	
gcdH	Sense	5' GGGGAATTCATGGCATTAAAAACAG 3'	c2X	1326	*EcoR*I	49
	Antisense	5' GGGTCTAGATTATTTTCCAACAGTAAAA 3'			*Xba*I	
***Edwardsiella tarda* outer membrane proteins**						
ETAE_3048	Sense	5' CCCGAATTCATGTATCTGATTGATTA 3'	32a	771	*EcoR*I	28
	Antisense	5' GGGCTCGAGTTATTTCCCCGGGAA 3'			*Xho*I	
ETAE_0960	Sense	5' GGGGAATTCATGAGTTTATTCATTTCT 3'	32a	333	*EcoR*I	12
	Antisense	5' GGGCTCGAGTTACAGCGCTTTCATC 3'			*Xho*I	
Danio rerio						
ucp2	Sense	5' CCCGAATTCATGGTTGGATTCAGAGCT 3'	32a	933	*EcoR*I	33
	Antisense	5' CCCTCTAGATTAAAGAGGAGTGTGCCAG			*Hind*III	
IL-1β	Sense	5' CCCGAATTCATGGCATGCGGGCAATAT 3'	32a	822	*EcoR*I	30
	Antisense	5' CCCGTCGACCTAGATGCGCACTTTATC 3'			*Sal*I	
bcl2	Sense	5' CCCGAATTCATGGCTAACGAAATTAGC 3'	32a	678	*EcoR*I	26
	Antisense	5' CCCGTCGACTCACTTCTGAGCAAAAA 3'			*Sal*I	
	Antisense	5' CCCGTCGACTTACACTCGAGGATTGAC 3'			*Sal*I	
CC-chemokine	Sense	5' CCCGAATTCATGCAGCTCAACCAGAA 3'	32a	417	*EcoR*I	14
	Antisense	5' CCCGTCGACTTAAGAAGAGGAGGTGG 3'			*Sal*I	
